# Impact of an Existential-Spiritual Intervention Compared with a Cognitive-Behavioral Therapy on Quality of Life and Meaning in Life among Women with Multiple Sclerosis

**DOI:** 10.18502/ijps.v15i4.4298

**Published:** 2020-10

**Authors:** Marzieh Hajibabaei, Bagher Kajbaf, Maryam Esmaeili, Mohammad Hossein Harirchian, Ali Montazeri

**Affiliations:** 1 Department of Psychology, Faculty of Education and Psychology, University of Isfahan, Isfahan, Iran.; 2 Psychosomatic Medicine Research Center, Tehran University of Medical Sciences, Tehran, Iran.; 3 Iranian Center of Neurological Research, Neuroscience Institute, Tehran University of Medical Sciences, Tehran, Iran.; 4 Population Health Research Group, Health Metrics Research Center, Iranian Institute for Health Sciences Research, Academic Center for Education, Culture and Research (ACECR), Tehran, Iran.; 5 School of Humanity Sciences, University of Science and Culture, Academic Center for Education, Culture and Research (ACECR), Tehran, Iran.

**Keywords:** *Cognitive-Behavioral Therapy*, *Existential-Spiritual Intervention*, *Meaning in Life*, *Multiple Sclerosis*, *Quality of Life*

## Abstract

**Objective:** Multiple sclerosis (MS) is a chronic neurological disease that could aggressively affect patients’ quality of life in most instances. This study aimed to compare the effectiveness of an existential-spiritual psychotherapy with a cognitive-behavioral therapy on quality of life and meaning in life in women with multiple sclerosis.

**Method** **:** A convenience sample of 43 women with multiple sclerosis participated in this quasi-experimental study. They were randomly assigned into 3 groups: an existential-spiritual intervention, a cognitive-behavioral intervention, and the control group. Participants were assessed for outcome measures (quality of life and meaning in life) at 3 points in time: pretest, posttest, and 5-months follow-up. The Multiple Sclerosis Quality of Life-54 (MSQOL-54) and the Meaning in Life Questionnaires (MLQ) were used as outcome measures. To compare outcomes among the study groups, repeated measures analysis of variance was performed.

**Results: **The results showed that while no difference was observed for the control group, scores for meaning in life improved significantly for existential-spiritual intervention and cognitive-behavioral therapy (p = 0.027, p = 0.039). Also, both mental (p < 0.001, p = 0.014) and physical (p = 0.001, p = 0.013) health dimensions of quality of life increased significantly in the 2 intervention groups. However, the results indicated that women in the existential-spiritual intervention group showed greater improvement in some aspects of meaning in life (search for meaning) and quality of life (role physical and role emotional, pain and energy) compared to women in the cognitive-behavioral intervention group. However, the latter group showed better improvements on 2 subscales (physical function and health distress).

**Conclusion: **Both existential-spiritual and cognitive-behavioral interventions can improve quality of life and meaning in life among women with multiple sclerosis. However, the findings suggest that although both interventions were effective, the existential-spiritual intervention resulted in more positive improvements in some aspects of meaning in life and quality of life.

Multiple sclerosis (MS) is the most inflammatory-demyelinating disabling disease of the central nervous system (CNS) (1-2) that generally occurs at age between 20 and 50 years (3).

Over 2 million people have multiple sclerosis worldwide (4).

 In Iran, the prevalence of MS has been estimated 100 in 100 000, which is considered to be one of the high-risk countries in Middle East (5). The ratio of women with MS is more than 3 times that of men (6).

Although MS has scarcely affected life expectancy, it can have several physical, psychological, and socioeconomic effects on individuals and societies (7-11). Also, MS has a destructive effect on patients’ quality of life (12).

While there is no definite treatment for MS, the existing therapeutic strategies are aimed at reducing the risk of relapses and possibly severe disability (13). However, a number of nontherapeutic strategies aim to improve quality of life. One such strategy is the use of logotherapy that roots from existential philosophical concepts (14) and was introduced by Frankl (1984). According to Frankl, every person has a unique task waiting to be fulfilled in life and it is that person's responsibility to actualize its meaning. Frankl also believes that one can transcend suffering if one has a reason to live. For Frankl (1984), ‘he who has a why to live for, can bear anyhow’ (p. 97) (15-16). Such evidence suggests that logotherapy can improve the meaning of life and may be effective in relieving the death anxiety caused by recurrent cancer (17). Also, it has been reported that logotherapy can reduce depression and demoralization in patients with breast and gynecological cancers (18).

Moreover, a number of investigators suggest that spiritual well-being is associated with a better mental health (19), overall health (20), and quality of life (21). In contrast, lack of spiritual well-being may result in depression, stress, anxiety, and lack of meaning and purpose in life (22). Spiritual well-being is defined as the benefits of the way individuals seek and express meaning and purpose and the way they experience their connectedness to the moment, self, others, nature, and to the significant or sacred (23). 

There is evidence that spiritual well-being exerts a substantial influence on psychosocial adaptation to MS (24). Thus, we assumed that augmenting existential–phenomenological psychology with spiritual themes may provide therapists a holistic and comprehensive approach. 

Cognitive–behavioral therapy (CBT) is a structured and combined psychological intervention that is used to identify the effect of patients’ experiences based on their belief system. CBT helps to reconstruct thoughts and change responses through new skills and behaviors. It has been increasingly used among patients with chronic diseases, including those with MS, to manage symptoms (25, 26) or improve psychosocial outcomes (27). CBT is specially applied to address MS-related complications; eg, major depressive disorder, or high levels of depressive and anxiety symptoms (28). Also, studies have shown that CBT may improve psychosocial adjustment (29) and quality of life (30).

Therefore, this study aimed to assess and compare the effect of existential-spiritual psychotherapy and cognitive-behavioral interventions on quality of life and meaning in life among women with MS. The selection of MS patients for this study was due to the fact that MS generally is an unexpected and sometimes debilitating disease; therefore, patients may be challenged by their thoughts on meaning in life and some essential beliefs and existential anxieties (31). 

## Materials and Methods

***Design***

This quasi-experimental study, with 1 control and 2 intervention groups, was conducted on a sample of women with MS in Tehran, Iran. Patients in interventions groups received 8 weekly sessions, while the control group received none. All participants were assessed at 3 points in time (pretest, posttest immediately after completion of intervention, and at 5 months follow-up).

***Participants***

A convenient sample of patients with multiple sclerosis who were members of Iranian MS Society or those who referred to an MS center of a teaching hospital was approached and randomly assigned into the study groups. According to results of a previous study, (32) we used the following formula to estimate the sample size: 


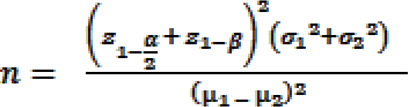


α = 0.05, β = 0.2, µ1 = 55.7 (mean score for quality of life, immediately after intervention), σ1 = 15.3, µ2 = 39.8 (mean score for quality of life, control group), σ2 = 17.4. 

Inclusion criteria were as follow: definitive MS diagnosis by neurologist; lack of major physical or mental comorbidity; literacy; age 18 years and older; physical ability for participating in sessions; at least 6 months from diagnosis of MS; and no simultaneous psychological interventions. Exclusion criteria were as follow: acute attack; 3 times absent or more; and unwillingness to continue participation. We used random assignment for dividing patients into 3 groups: existential-spiritual intervention (n =16) (group1), cognitive-behavioral intervention (n = 16) (group 2), and control (n =16) (group 3).

***Intervention***

1. The existential-spiritual intervention was a comprehensive psychotherapy based on the Holy Quran, spiritual themes, and Frankele’s logotherapy (Table 1) (33). The intervention included 8 sessions on life skills improvement, coping self-awareness, meaningfulness, freedom, believing in God's blessing, eternal being, and faithfulness to moral values.

2. The cognitive-behavioral group therapy was based on the White therapeutic guideline for 8 weekly sessions (Table 1) (25). The sessions consisted of reviewing current problems, setting the goals, debating about common cognitive distortions, relationship between thoughts, emotions and behaviors, problem-solving, supportive and opposite evidences and relaxation techniques (34).

***Measures***

The following questionnaires were used for data collection:

1. Demographic and clinical information: A self-designed questionnaire was used to collect demographic characteristics (age, marital status, education, and occupation) and clinical information (duration of MS and hospitalization).
2. Multiple Sclerosis Quality of Life (MSQOL-54): The MSQOL-54 was used to measure quality of life. It has two main components: mental health and physical health components. The MSQOL-54 was developed by Vickery et al in 1995. The scores on each component or subscale can range from 0 to 100, where the higher scores indicate better conditions (35). The psychometric properties of the Persian version of MSQOL-54 are well documented (36).
3. Meaning in Life Questionnaire (MLQ): This 10-item self-report scale measures two dimensions: presence of meaning and search for meaning. The MLQ was developed by Steger et al in 2006. Each item is rated on a seven-point Likert scale ranging from absolutely true to absolutely untrue (37, 38). The Persian version of MLQ proved to be valid (38).

***Analysis***

In this study, analysis of variances and chi-square were used to compare the baseline differences among the 3 groups (two interventions and one control groups). Also, one-way analysis of variance was used to contrast between dependent variables between the 2 intervention groups at posttest and follow-up assessments. Changes from baseline to posttest and follow-up were analyzed through repeated measures among the 3 groups.

## Results

***Patients’ Characteristics***

A total of 43 patients were entered into the study. There were no significant differences among groups with regards to demographic characteristics (age, education, occupation, and marital status), and clinical status (duration of MS and hospitalization) at baseline. Also, no differences were seen between quality of life and meaning in life scores among the 3 groups (Table 2). 

***Quality of life***

Role physical (p = 0.006), role emotional (p = 0.036), pain (p < 0.001), emotional well-being (p = 0.007), energy (p = 0.006), health perception (p = 0.006), overall quality of life (p = 0.003), mental health component (p < 0.001) and physical health component (p = 0.001) improved significantly after existential-spiritual intervention.

Physical functioning (p = 0.007), emotional well-being (p = 0.023), health perception (p = 0.011), health distress (p = 0.036), overall quality of life (p = 0.01), mental health component (p = 0.014) and physical health component (p = 0.013) improved significantly after CBT (Table 3).

No significant impact of existential-spiritual intervention was found in physical function, cognitive function, social and sexual function, and health distress. Also, cognitive-behavioral therapy did not have a significant impact on role physical and role emotional, cognition, social and sexual function, energy, and pain.

***Meaning in Life***

After existential-spiritual intervention, scores of meaning in life (p = 0.027), presence of meaning (p = 0.030), and search for meaning (p = 0.045) increased significantly. Among CBT group therapy, meaning in life (p = 0.039) and presence of meaning (p = 0.025) improved significantly. No significant changes were observed in the control group at 3 assessments (Table 4).

**Table1:** The Content of the Existential-Spiritual Intervention and the Cognitive-Behavioral Therapy Protocol*

**Session**	**Title**	**Objectives**	**Content**
	Existential-Spiritual Intervention		
1	Motivation for changing and life skills training	Gain the trust of the group, familiarity with existential- spiritual psychotherapy life skills improvement (1st part)	Pretest, introducing the course, and a brief discussion on barriers to change, Identification of characteristics, abilities, and goals, description of importance of life skills such as self-awareness, discussion about causes and side effects of MS providing homework
2	Being purposeful and Coping strategies	Goal setting, identify cognitive distortions, life skills augmentation (part 2)	Misconceptions about MS, prioritizing the goals, discussion about active coping strategies, stress management, anger control and problem solving, providing homework
3	Responsibility in life and freedom challenge, fear of death	Death awareness, decreasing the death anxiety	Discussion about relationships' problems, spiritual- religious coping, believe in God's blessing, believe eternal being providing homework
4	Search for meaning	Self-transcendence, self- detachment, planning for goals	Short-term, intermediate, and long-term goals, acquiring moral values, providing homework
5	Values in cultural perspective	Explanation of creative values, empirical values and attitude values	Setting new goals regarding to the values, for example, working, love and art, pain and suffering, providing homework
6	Authentic life style and relationships	Improvement the communications alignment with goals	Exploration of four relationships: being with me, god, others, and nature, providing homework
7	Revise the define of suffering	New perspective to problems	Review the sufferings and discussing any losses and gains, providing homework
8	Review	Maintenance of acquired skills	Discussion about any changes that participants see in themselves as a result of sessions. posttest
	Cognitive-Behavioral Therapy		
1	Cognitive and behavior	Presenting the essentials of cognitive-behavioral model	pre-test, introducing the members of the group and, the patients' expectations from treatment, identifying current problems, receiving feedback, Discussion about causes and side effects of MS and providing homework
2	Updating and reviewing the mood	Preparing a periodic summary	Forming a relationship with the past session, homework review, discussion of items on the agenda, getting feedback, and providing related tasks
3	Automatic thoughts	To identify automatic thoughts,	explaining the patients about the automatic thoughts and evaluating the automatic thoughts during the session relative to the MS illness, teaching the relaxation, and providing a homework
4	Identifying the emotions	Differentiating between automatic thoughts and excitement	Discussion about primary emotions, time management and revising activities by using the activity table
5	Cognitive errors	Identifying the common cognitive errors about MS	Assessment of automatic thoughts and training the answers to automatic thoughts and providing assignments
6	Intermediate beliefs	Identification and correction of intermediate beliefs	Using the Socratic questioning technique, assessing the gains and difficulties of a belief and the behavioral test, cognitive conceptualization and proving the assignment
7	Fundamental beliefs	Identifying fundamental beliefs and reviewing their functions	correcting fundamental beliefs using the fundamental belief worksheet and the technique of reviewing the supportive and contrasting evidence, providing the assignment
8	An overview of sessions	Complete the treatment, and relapse prevention	Problem-solving training, conclusion of treatment, post-test

* Adapted from (34)

**Table 2:** The Baseline (Pretest) Quality of Life (MSQOL-54) and Meaning in Life (MLQ) Scores among the Study Groups

	**Group1**	**Group2**	**Group3**	
	**Mean (SD)**	**Mean (SD)**	**Mean (SD)**	**P**
**MSQOL-54**				
Physical function	73.43 (22.4)	59.61 (17.6)	64.64 (23.9)	0.230
Role physical	50.00 (40.8)	34.61 (33.1)	51.78 (42.1)	0.462
Role emotional	47.91(43.8)	46.15 (42.0)	52.38 (46.6)	0.930
Pain	68.64 (22.7)	58.33 (22.9)	64.64 (19.7)	0.456
Emotional well-being	56.00 (21.3)	61.00 (18.6)	51.42 (17.2)	0.459
Energy	47.00 (19.6)	49.53 (16.7)	45.14 (11.1)	0.785
Health perception	66.87 (16.2)	57.30 (20.3)	55.35 (18.3)	0.190
Social functioning	40.62 (17.7)	32.69 (18.1)	35.11 (17.0)	0.464
Cognitive functioning	65.62 (26.5)	56.15 (27.3)	60.00 (29.0)	0.651
Health distress	80.62 (20.0)	68.07 (23.5)	66.78 (22.1)	0.171
Sexual functioning	68.75 (23.8)	63.88 (32.2)	73.15 (26.9)	0.776
Overall QoL	69.47 (17.1)	64.22 (21.6)	58.19 (23.1)	0.363
Mental health composite	59.64 (21.6)	56.38 (18.6)	55.15 (17.6)	0.833
Physical health composite	60.91 (14.1)	51.34 (12.4)	54.46 (13.7)	0.159
**MLQ**				
Presence of meaning	27.06 )7.1)	24.00 (7.0)	25.07 (7.4)	0.513
Search for meaning	30.00 (4.5)	26.30 (6.8)	28.28 (6.2)	0.253
Total meaning in life score	57.06 (11.2)	50.30 (9.5)	53.35 (12.6)	0.281

**Derived from one-way analysis of variance**

Group 1: existential-spiritual intervention

Group 2: cognitive-behavioral therapy

Group 3: control

**Table 3:** The Results Obtained from Repeated Measures Analysis for MSQOL-54 Scores for the Study Groups

	**Pretest**	**Posttest**	**Follow-up**	
	**Mean (SD)**	**Mean (SD)**	**Mean (SD)**	**p**
**Group1 (n=16)**				
Physical functioning	73.43 (22.4)	72.81 (23.1)	73.12 (23.2)	0.876
Role physical	50.00 (40.8)	62.50 (35.3)	68.75 (35.9)	0.006
Role emotional	47.91 (43.8)	66.66 (40.3)	68.75 (41.2)	0.036
Pain	68.64 (22.7)	81.77 (16.8)	82.81 (16.4)	<0.001
Emotional well-being	56.00 (21.3)	71.75 (11.5)	70.75 (13.2)	0.007
Energy	47.00 (19.6)	63.25 (13.5)	61.25 (14.5)	0.006
Health Perception	66.87 (16.2)	76.56 (15.8)	76.87 (14.9)	0.006
Social functioning	40.62 (17.7)	34.37 (13.5)	35.93 (12.8)	0.236
Cognitive functioning	65.62 (26.5)	70.93 (18.8)	72.18 (19.0)	0.186
Health distress	80.62 (20.0)	88.43 (12.6)	89.06 (12.9)	0.062
Sexual functioning	68.75 (23.8)	78.48 (20.2)	79.55 (16.3)	0.54
Overall quality of life	69.47 (17.1)	80.67 (14.7)	81.87 (14.7)	0.003
Physical health composite	60.91 (14.1)	68.04 (11.3)	68.69 (10.6)	0.001
Mental health composite	59.64 (21.6)	72.25 (16.0)	83.18 (17.9)	<0.001
**Group2 (n=12)**				
Physical functioning	59.61 (17.6)	74.23 (15.9)	73.75 (16.5)	0.007
Role physical	34.61 (33.1)	53.84 (41.8)	52.08 (43.2)	0.193
Role emotional	46.15 (42.0)	64.10 (39.5)	61.11 (39.7)	0.189
Pain	58.33 (22.9)	68.07 (21.9)	68.47 (22.8)	0.191
Emotional well-being	61.00 (18.6)	70.46 (17.7)	70.33 (18.5)	0.023
Energy	49.53 (16.7)	58.76 (19.3)	58.33 (20.1)	0.150
Health perception	57.30 (20.3)	72.69 (16.4)	71.66 (16.5)	0.011
Social functioning	32.69 (18.1)	29.48 (15.4)	31.25 (14.7)	0.470
Cognitive functioning	56.15 (27.3)	70.76 (26.5)	69.16 (27.0)	0.079
Health distress	68.07 (23.5)	82.30 (15.4)	81.25 (15.6)	0.036
Sexual functioning	63.88 (32.2)	84.16 (19.8)	75.92 (33.7)	0.240
Overall quality of life	64.22 (21.6)	76.78 (13.6)	77.90 (12.3)	0.010
Physical health composite	51.34 (12.4)	63.75 (13.7)	62.74 (14.0)	0.013
Mental health composite	56.38 (18.6)	71.77 (17.6)	77.85 (20.4)	0.014
**Group3 (n=12)**				
Physical functioning	64.64 (23.9)	65.00 (24.9)	66.25 (18.2)	0.998
Role physical	51.78 (42.1)	55.76 (41.0)	56.25 (24.1)	0.754
Role emotional	52.38 (46.6)	53.84 (48.1)	55.55 (49.9)	0.674
Pain	64.64 (19.7)	65.25 (20.3)	66.80 (20.4)	0.339
Emotional well-being	51.42 (17.2)	52.61 (17.3)	52.66 (15.8)	0.842
Energy	45.14 (11.1)	46.46 (10.3)	47.00 (8.3)	0.870
Health Perception	55.35 (18.3)	57.69 (16.7)	58.33 (16.6)	1.000
Social functioning	35.11 (17.0)	35.89 (15.3)	38.88 (11.4)	0.339
Cognitive functioning	60.00 (29.0)	59.61 (30.1)	66.66 (25.4)	0.140
Health distress	66.78 (22.1)	69.23 (21.0)	71.25 (14.3)	0.709
Sexual functioning	73.15 (26.9)	69.79 (21.3)	75.00 (24.0)	0.356
Overall quality of life	58.19 (23.1)	60.15 (24.2)	66.24 (26.5)	0.223
Physical health composite	57.24 (12.5)	56.69 (12.7)	56.92 (8.4)	0.841
Mental health composite	55.15 (17.6)	53.48 (20.1)	59.83 (17.1)	0.490

Group 1: Existential-spiritual intervention

Group 2: Cognitive-behavioral therapy

Group 3: Control

**Table 4:** The Results Obtained from Repeated Measures Analysis for Meaning in Life Scores for the Study

	**Pretest**	**Posttest**	**Follow-up**	
	**Mean (SD)**	**Mean (SD)**	**Mean (SD)**	**P**
**Group 1 (n=16)**				
Presence of meaning	27.06 (7.12)	29.93 (4.55)	31.12 (2.98)	0.030
Search for meaning	30.00 (4.51)	31.25 (4.18)	32.31 (2.15)	0.045
Meaning in life	57.06 (11.23)	60.75 (7.18)	63.43 (4.51)	0.027
**Group2 (n=12)**				
Presence of meaning	24.00 (7.05)	26.53 (7.04)	28.41 (6.70)	0.025
Search for meaning	26.30 (6.82)	27.15 (7.17)	29.16 (5.57)	0.299
Meaning in life	50.30 (9.56)	53.84 (12.00)	57.58 (12.06)	0.039
**Group3 (n=12)**				
Presence of meaning	25.07 (7.48)	26.76 (7.072)	26.83 (6.32)	0.297
Search for meaning	28.28 (6.26)	29.23 (4.83)	28.25 (5.81)	0.365
Meaning in life	53.35 (12.60)	56.00 (11.04)	55.08 (11.34)	0.705

Group 1: Existential-spiritual intervention

Group 2: Cognitive-behavioral therapy

Group 3: Control

## Discussion

The study results indicated that an existential-spiritual intervention could improve some aspects of mental and physical health in women with MS.

Similar observations have been reported in prior studies that examined the logotherapy effect on quality of life among patients with MS (39, 40). Moreover, a number of studies showed similar results for other diseases. For instance, among community-dwelling adults with cardiovascular diseases who took part in a 1-month individualized spirituality-based intervention and demonstrated a relatively fair increase in overall quality of life (41). Similarly, logotherapy was effective in improvement of quality of life of adolescents with terminal cancer (14).

In this study, the findings showed that cognitive-behavioral therapy could improve some aspects of quality of life in this population. Likewise, in parallel with this study, some investigators proved that cognitive-behavioral therapy (CBT) improves mental health and quality of life in MS patients (26, 42 and 43). The effectiveness of CBT could be related to its several properties, including modification of thoughts and identification of emotions and cognition errors, which result decreasing stress and improving mental health. However, these results require further evaluation considering that other factors could also affect quality of life in patients with MS. 

Moreover, we observed that existential-spiritual therapy could improve life’s meaning and its components. This result was expected because the main goal of this intervention was motivating patients to search for the meaning of life according to their values. Also, ‘meaning’ is an element of spirituality that appears to be a more common concept that can exist in religious or nonreligious individuals (44); thus, improvement of meaning, will result in better spirituality. Similarly, spiritual well-being is related to less perceived illness and psychological distress (24), as well as better coping and psychosocial adjustment (20, 24). Also, Nsamenang emphases a multidimensional attitude, including promotion of spiritual well-being for addressing physical or psychological status of MS patients (45).

Puchalski and Romer (2000) defined spirituality as “a concept that allows a person to experience transcendent meaning in life.” (46). Our finding is consistent with those of prior researches. Kang et al showed that logotherapy could decrease suffering and increase the meaning in life among adolescents with lethal cancer (47) and can be applied for adolescents to prevent existential pain (14). Also, according to Breitbart, a comprehensive intervention that includes spiritual features to existential distress, demoralization, and loss of meaning should be developed and implemented for patients with advanced cancer (44).

The study results showed that CBT can improve meaning in life, which is consist with a study showed that cognitive behavioral therapy increases spiritual well-being significantly among the elderly mourners (48). This could result from the CBT effects on reconstructing the thoughts, changing behavior, and new objectives for life. The findings indicated that some aspects of quality of life, such as physical functioning social functioning, and sexual function, did not change among women in the 2 intervention groups; this may have several reasons: it can be due to small sample size and the fact that people with different cultural backgrounds may respond differently to issues such as social functioning or sexual functioning. 

## Limitation

This study has some strengths and limitations. To some extent, the quasi-experimental design of the study with a comparison group and the use of well-known standard measures to assess the quality of life and meaning in life could be regarded as strengths. However, the study had 2 main limitations. First, participants were recruited through convenience sampling from patients who were members of Iranian MS Society or those who referred to an MS Center of a teaching hospital. Therefore, our findings may not be generalized to all women with MS. Secondly, we did not measure other psychological variables that may mediate associations with quality of life and meaning in life. Thus, further studies are needed to explore factors that affect quality of life among women with MS.

## Conclusion

Both existential-spiritual and cognitive-behavioral interventions can improve quality of life and meaning in life among women with multiple sclerosis. However, the findings suggested that although both interventions were effective, the existential-spiritual intervention resulted in more positive improvements in some aspects of meaning in life and quality of life. In general, it seems that both existential-spiritual intervention and cognitive-behavioral therapy can be helpful to improve quality of life and meaning in life among women with MS. The interventions also can help women to overcome their problems and adjust themselves to the disease and its consequences.
